# Assassin snails (*Anentome helena*) as a biological model for exploring the effects of individual specialisation within generalist predators

**DOI:** 10.1371/journal.pone.0264996

**Published:** 2022-03-14

**Authors:** Boris W. Berkhout, Andrew Morozov

**Affiliations:** 1 Department of Genetics and Genome Biology, University of Leicester, United Kingdom; 2 Department of Mathematics, University of Leicester, United Kingdom; 3 Institute of Ecology and Evolution, Russian Academy of Sciences, Moscow, Russia; University of Waikato, NEW ZEALAND

## Abstract

Quantifying feeding behaviour of generalist predators at the population and individual levels is crucial for understanding the structure and functioning of food webs. Individual predator/consumer feeding niches can be significantly narrower than that of the population across animal taxa. In such species, the population of a generalist predator becomes essentially an ensemble of specialist individuals and this often highly affects the dynamics of the prey-predator interactions. Currently, few experimental systems exist that are both easily technically manipulated in a lab and are reliable to accurately assess effects of individual specialisation within generalist predators. Here we argue that a freshwater predaceous snail, *Anentome helena* (also known as an ‘assassin snail’), is a convenient and reliable experimental system to study feeding of a generalist predator on multiple food types which exhibits well-pronounced specialisation of foraging individuals. Using *A. helena* we experimentally test: (i) how relative prey abundances in the environment affect the feeding patterns, (ii) whether the feeding patterns are consistent over the duration of the experimental period, and (iii) compare the feeding niche breadth of individuals to that of the laboratory population. By offering four different prey snail species, at a range of relative abundances, we show that there are consistent patterns in feeding. Importantly, the consumption of each prey was independent of the relative abundance at which they were present. Individual predators showed selectivity to a particular prey, i.e. the population of assassin snails seems to be formed of individuals that specialise on different prey. Our findings would contribute to the recent revision and the ongoing debate on the classification of predator species into generalists and specialists.

## Introduction

Foraging ecology explores how consumer resource uptake is shaped by the biotic and abiotic environment. The knowledge of foraging behaviour of generalist predators is crucial for understanding the functioning of food webs, community dynamics and explaining ecosystem biodiversity [1–3]. While great progress has been made, a unified theory of food selection by generalist predators using a large number of resources has not yet emerged [4–6]. Foraging of a predator consuming multiple food types is often described using some theoretical rationale (mathematical modelling) [4, 7, 8]. Alternatively, one can extrapolate a mathematical or statistical model obtained from simple foraging scenarios with few prey types to a situation where the predator faces many available types of prey [5]. Finally, foraging of a generalist predator can be modelled using deduction based on a set of reasonable assumptions [9]. It is to be stressed that for further progress empirical verification of existing theories of a consumer feeding on multiple resources is essential especially with regards to variability with predator populations.

Generalist predators are those that feed on a range of prey species. However, some obstacles still exist in exploring and understanding the foraging behaviour of generalist predators. First, the perception of resources by a predator can differ from human definitions. Biologically separate species could be treated as a similar resource, or members of the same species could be seen as different [10]. If different palatable prey types are not perceived as distinct by a predator it is expected that they are consumed proportionally to their relative abundance, because there will be no selection of one prey over the other. In such cases, the observed pattern can be described as ‘random prey selection’ or ‘random feeding’ [6]. Second, relative prey profitability may vary over time. For example, differences in nutrient content may drive predators to preferentially feed on different prey depending on their requirements. Such an example of change in diet is seen during the development of *Nucella emarginata* [11]. Third, populations are rarely homogeneous in terms of their feeding behaviour, leading to inter-individual variation in feeding patterns [12]. Indeed, individuals within a predator population often differ in terms of their diets and in some cases are best described as a collection of individual specialists [13]. This can be due to morphological, age, and sex differences. Other factors, such as behavioural or cognitive traits of individual predators, may have a profound effect on diet variation of individuals as well [12, 13]. For example, high consistency in individual differences in food intake of African catfish (*Clarias gariepinus*) was correlated with individual differences in growth [14]. In red knots individual diet preferences caused differences between individuals in their physiology [15]. Further, an increase in intra-specific competition led to greater individual specialisation of individual diet of the population of the Eurasian perch [16] and a tropical seed-eating bird *Pyrenestes ostrinus* [17]. We can then define an ‘individual specialist’ as a predator having a feeding niche that is significantly narrower than that of the population [18], and the feeding niche of a population as the sum of individual niches. Comprehension of such individual specialisation of predators is important for understanding ecosystems stability, success and failure of biological invasions, and development of adaptive speciation [19]. Finally, generalist and specialist predator definitions are not always clear-cut [19], for instance, in Eurasian perch (*Perca fluviatilis*) niche breath, and thus levels of specialisation, varies with habitat [20].

Apart from some well-studied systems [11, 21, 22], much of the work on individual specialisation has been conducted in vertebrates. To gain a more comprehensive understanding a wider range of species should be investigated. Some of these questions have been successfully addressed in marine molluscs [11, 21, 22], but rarely in their fresh-water counterparts [23]. Additionally, mollusk ecology overall has received only limited scientific interest compared to the high biodiversity of this group [24]. The number of described species of mollusks—around 100,000—is second only to the arthropods [25]. Additionally, molluscs occur in marine, fresh water and terrestrial habitats and display a range of trophic and ecological roles [25]. This means that considerable knowledge could be gained from studying this group of consumers. Here, we suggest the freshwater predaceous snail *Anentome helena* as a convenient system to further explore foraging patterns of a generalist predator feeding on multiple resources, and the potential for individual feeding niche separation.

*A. helena* (von dem Busch in [26], Nassariidae, previously also known as *Clea helena*) is a fresh water snail [27, 28]. It actively hunts other freshwater snails and worms, but also scavenges on dead fish [29, 30]. It subdues snail prey with its foot and then consumes the prey through the prey’s aperture [31]. For this reason it is popular in the aquarium trade [32], as a species that preys on ‘pest snails’ and is known under a wide range of names including ‘assassin snail’, ‘snail-eating snail’, ‘killer snail’, and ‘bumble bee snail’ [33]. *A. helena* is native to many areas in Southeast Asia, and recently range expansion has been reported, showing its invasive potential [29]. Surprisingly, its foraging behaviour has not accurately been addressed experimentally.

In this paper we use *A. helena* to test a number of hypotheses. First, we test how prey relative abundances affect feeding electivity of *A. helena* snails. Second, we test whether any patterns in electivity of prey are consistent over time. Third, we compare diet breadth of individual *A. helena* to that of the laboratory population. Finally, based on these results we evaluate the suitability of *A. helena* as a system to study predator-prey interactions, specifically regarding the niche breadth of individual predators. We expect that individuals exhibit a consistent, but narrower feeding niche than the population as a whole. This will result in stable differences in feeding electivity.

## Materials and methods

### Animal husbandry

All snails stocks (predator and prey) were obtained from a local aquarium shop (Leicester Aquatics). In experiments predators and prey from a mixture of first and second (lab bred) generation snails were used. Snails used in the experiment were never used for breeding afterwards.

**Fig 1:**
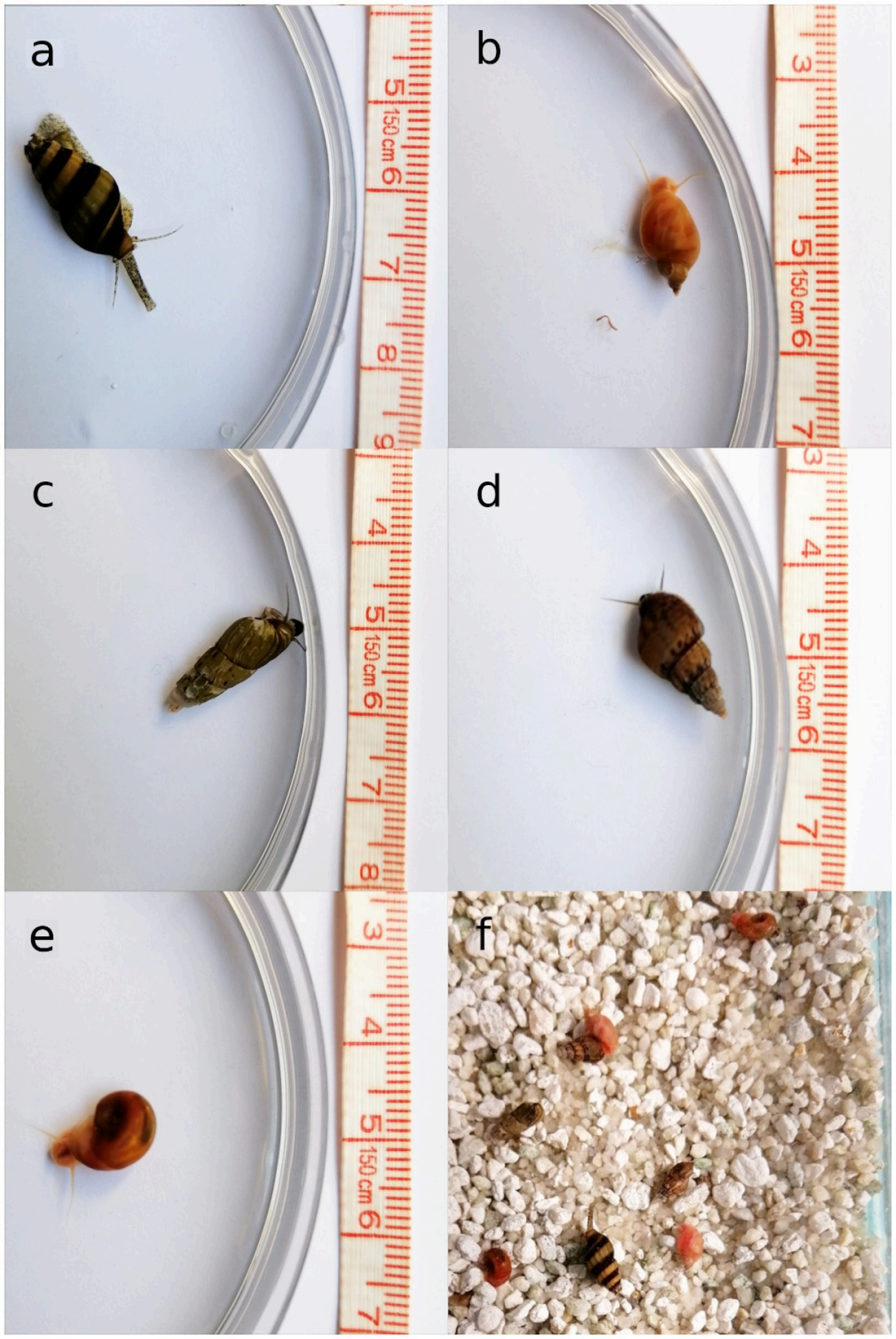
Overview of snail species used in the experiment. Snail species used in the experiment with their characteristic body shapes. Prey snail species used in the experiment with *A. helena* (a) include: Pond snails (*Lymnaea* sp.; b), Trumpet snails (*Melanoides tuberculata*; c), Quilted Melania snails (*Tarebia granifera*; d), and Ramshorn snails (*Planorbella* sp.; e). Top-down view of a typical experimental compartment (f) containing the predator and the four types of prey (in equal relative abundances). Note that panel f is on a different scale from the other panels, roughly 2:1. Photos credit: Oksana Gonchar (University of Leicester, UK).

*A. helena* assassin snails (Fig 1a) were housed in groups of 10–20 in stock tanks (approximately 20 x 30 x 20 cm) aerated through a biological filter before trials. Each tank contained a layer (1–2 cm) of coarse sand to allow *A. helena* to bury themselves [31] and was filled with reconstituted water (de-ionized water with Tropic Marin Seasalt). Conductivity levels were kept between 2.0 and 2.5 mS/m as this helps suppress bacterial infections in aquatic animals [34]. All snails were kept under a 12h:12h light:dark regime. The stock and experimental tanks were kept in a climate room set to 25°C (±1°C). Water was changed approximately every three weeks to ensure good water quality.

During the pre-experimental period, *A. helena* in each tank were fed by placing a cube of frozen blood worms (Chironomidae) at the bottom of the stock tank. *A. helena* snails readily consumed this and were fed on blood worms for at least three weeks before the start of the experimental trials. This was done to remove any pre-existing behavioural adaptation (e.g. more efficient handling), as well as to prevent the development of a preference for one prey species. Preferences can develop over time in some predaceous snails [11, 35].

Due to limited research on *A. helena* in its natural habitat, little is known about the diet in the wild. We therefore picked prey species that are common in the aquarium trade and originating from Southeast Asia; Ramshorn snails (*Planorbella* sp.), Malaysian Trumpet snails (*Melanoides tuberculata*), Pond snails (*Lymnaea* sp.), and Quilted melania snails (*Tarebia granifera*; Fig 1b–1e). These were also selected for their different morphologies and behaviours, because it is important that the different prey species are perceived as different by the predator [6]. In brief, Ramshorn snails are planispiral and are active movers. Pond snails also actively move, but have a spiralling shell. The other two species are less active and are more likely to bury themselves. Trumpet snails have a slender, pointed shell, and Quilted melania snails have a broad, pointed shell.

Prey snail species were maintained in a similar way to *A. helena* snails with some differences. The tanks used were the same, but gravel (ø 5–20 mm), instead of sand, was added to the bottom to serve as a biological filter. A small amount of washed, crushed sea shells was added, to stabilise pH and calcium availability for growing snails. Aeration was supplied through air stones and snails were fed on a mixture of lettuce, and commercial fish food (TetraMin Tropical Flakes). Prey selection by predators can be based on nutrient compositions of prey diet [36, 37], so all prey species were fed on the same diet. Prey snails were kept in higher densities per tank (up to 40–50 individuals per tank) and water was changed every two weeks. All snail species fared well under these conditions as breeding and maturation were seen continuously.

### Experimental setup

For feeding experiments, four large tanks (60 x 40 x 40 cm) were subdivided with dividers into 10 (2 rows of 5) compartments each. Each compartment measured ∼11 by 19 cm and was filled with 6 cm of water (total volume of ∼1.25 L). Circa 1.5 cm of depth (250 mL) of white, fine (ø 5–7 mm) crystalline gravel was added to each compartment to allow for natural burrowing behaviour of snails (Fig 1f). With 8 prey snails per compartment, this resulted in a prey density of 640 m^−2^, which falls within the range of densities in the natural habitat [38, 39]. Water exchange between compartments within a tank was possible through small (ø 3 mm) holes in the dividers, while ensuring both prey and predaceous snails were confined within compartments. This allowed for maximum feeding as predaceous snails can be encouraged to feed by the odours of their conspecifics feeding [40]. Two central compartments were reserved for a biological aquarium filter and a thermometer. Eight replicates could be run simultaneously in one tank.

Five different experimental treatments were carried out. Each treatment used 8 prey individuals out of the four prey species. Initial trials were carried out with equal relative prey abundances (2 Ramshorn: 2 Trumpet: 2 Pond: 2 Quilted melania; n = 17). Later, four different prey relative abundance treatments were tested in haphazard order (details on starting dates available in the data set); 1:2:1:4 (n = 31), 1:3:2:2 (n = 10), 2:1:4:1 (n = 12), and 4:2:1:1 (n = 17). Not all treatments could be run simultaneously, because of availability of prey snails and space constraints. Different numbers of replicates for the different treatments were obtained due to prey snail availability. A single treatment was tested in a tank at the same time. During most trials, one or two control treatments with the same relative abundances of prey snails, but without *A. helena* snails were used to investigate the background prey mortality. It was also checked that all prey species were consumed, i.e. that all prey species were recognised as such, and whether there were any differences in total consumption between treatments as this could have implications for the electivity. To standardise hunger levels between *A. helena* snails, they were starved between 3 and 7 days before trials [41]. For practical reasons they could not always be starved for the same amount of time. Within a trial starvation times were always equal.

Feeding by each *A. helena* snail was tested for 14 consecutive days. For one chosen treatment (1:2:1:4), we ran trials for 28 days, to monitor potential shifts in feeding behaviour over the duration of the experimental period. The consumption by each *A. helena* snail was recorded every 24 hours by counting empty shells of prey snails. Then, all prey snails and empty shells were removed and replaced with new prey snails at the same starting relative abundances. Live prey were returned to holding tanks and fed. These prey snails could then be used again in the same trial (i.e. the same treatment with the same *A. helena* snails), in compliance with the 3R’s (‘Replace, Reduce, Refine’ [42]). All prey snails were of similar size (100–250 mg wet weight) at the start of a trial to minimise the effect of prey size on prey selection by *A. helena* snails. Prey snails were approximately half the wet weight of *A. helena* snails (350–600 mg wet weight; similar range across treatments) used in the experiment. This amounted to small (10–15%) differences in length within species. All predators were offered a similar range of prey sizes to make food abundance as equal as possible. Preliminary experiments had shown that this leads to a steady feeding rate, whilst minimising the consumption of more than one prey by a single *A. helena* snail in a day and consequently affecting prey relative abundances in a given compartment. At the end of each trial all prey and predators were removed and not used again.

### Statistical software and analyses

All calculations and statistical analyses were carried out in R [43] using R-studio [44]. GLMM’s (Generalised linear mixed models) were run in glmmTMB [45]. Comparisons between factorial treatments were done with multicomp [46] with a Tukey correction, and model fits were compared using the anova() function [47]. The Anova() [48] function was used to extract main effects from models with multiple factorial levels. For all models, the appropriateness of the model fit to the data was tested using the DHARMa package [49]. The output of these is reported in the supplementary material (analysis script; [50]). Figures were created using the packages ggplot2 [51], and png [52]. For data handling reshape2 [53], purrr [54] and dplyr [55] were also used.

### Total feeding and feeding preference

Only *A. helena* snails that fed at least 3 times during the two week experimental period were included in the analyses. The total consumption per *A. helena* was compared between treatments to test for the effect of treatment on willingness to feed. Consumption was compared using GLMM’s. Throughout the analyses Poisson and Conway-Maxwell-Poisson error distributions were used, based on appropriateness and model fit.

For the overall preference of each *A. helena* snail the Manly-Chesson *α* electivity index was calculated for each prey species each day [56, 57], according to the following simple expression

$$\begin{array}{c} \alpha_{i}=\frac{r_{i}/p_{i}}{\sum_{i}r_{i}/p_{i}}, \end{array}$$ (1)

where *r*_*i*_ is the proportion of prey item *i* consumed by the predator and *p*_*i*_ is the proportion (relative abundance) of the same prey type in the treatment. Although it is hard to statistically show absolute prey preferences using this index [58], it is possible to use this index to rank prey species and compare their rankings between treatments [58, 59].

For each *A. helena* snail, the electivity index for each experimental day on which it fed was calculated and averaged over all feeding days (results were similar to calculating the electivity index over all days combined; see S1 Fig in [50]). When selecting the prey type most consumed by an individual predator, ties were broken at random using the package nnet [60]. Within treatments the total consumption of prey species were compared with GLMM’s to determine potential electivity of prey species. A full model was constructed and compared to reduced models (see above). Models always included compartment nested within tank and predator ID as random effects to account for different experimental blocks and repeated measures. Additionally, it was tested whether prey species were more consumed than expected under ‘random feeding’. For this the Manly-Chesson *α* for each prey type was calculated and plotted with 95% confidence intervals (CI; [61]). If in these plots the CI crosses the expected-consumption line (1/number of prey types = 0.25, i.e. random feeding), there is no selection for this prey type [56]. This takes into account any differences in relative abundance of prey (see Eq 1 [56, 57]). If the CI falls completely above this line, there is ‘positive selection’ for this prey type, if the CI falls completely below this line there is ‘negative selection’—avoidance—of this prey type. When comparing between treatments, only the first two weeks of the 1:2:1:4 are used to allow for direct comparison between treatments. Both these measures (total consumption, deviation from random feeding) were also compared between the four different weeks for the 1:2:1:4 treatment using GLMM’s with ‘experimental week’ as a fixed effect.

### Diet breadth

Diet breath of individual predators was compared to that of the population using Petraitis’ index *W* [62]. This index gives the likelihood ratio of the observed diet of the individual (*i*) against the population, is suitable for discrete consumption, and its statistical properties are known [18, 62]. This standardised index ranges from 0 to 1 and quantifies the deviation of an individuals diet from that of the population. So, a 1 indicates a complete overlap with the population diet and values closer to zero indicate increasing values of specialisation. In this study, we present the distribution of Petraitis values using a histogram for each treatment. Using the histogram, for each snail it was calculated whether the diet significantly differed from that of the population. Petraitis *W*_*i*_ values and significance were calculated using the RInSp package [63].

## Results

### Total consumption of snails and non-predatory mortality of prey snails

**Fig 2:**
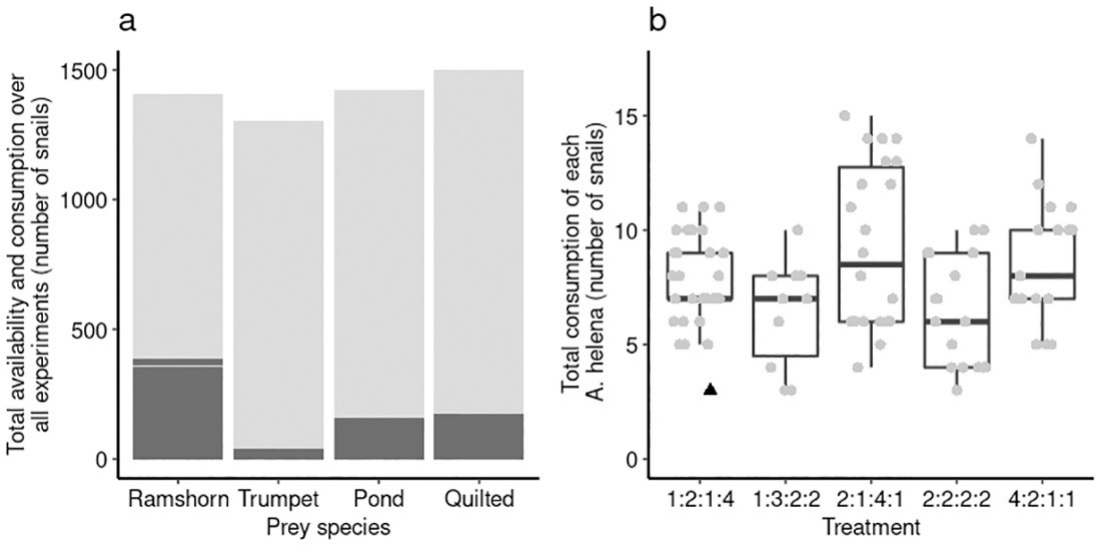
Total prey consumption overall and per treatment per predator. Total consumption of each prey species by *A. helena* (n = 97) over all treatments (a) and total consumption of prey snails at each treatment over the 14-day experimental period (b). All prey types were regularly consumed, however different numbers were consumed (dark grey: consumption; light grey: abundance). In (b) prey species are respectively, Ramshorn snails, Malaysian Trumpet snails, Pond snails, and Quilted melania snails. Box-plots with interquartile ranges and median, whiskers are up to the most extreme value within 1.5 times the interquartile range from the interquartile range. Individual points represent individual predators, outliers are indicated by triangles.

Only seven prey snails died in the control treatments (background mortality) over 121 experimental days (0.06 per day; n = 22 control trials). A total of 778 prey snails were predated upon over 1485 experimental days (0.52 per day; Fig 2a). There were no significant differences between the different prey species in background mortality (GLMM with treatment as random effect *χ*^2^ = 1.824, *P* = 0.610), removal of the random effect did not affect model fit (*χ*^2^ ≈ 0, *P* ≈ 1). The model had a good fit to the data (analysis script [50]). The background mortality of prey snails was thus considered negligible and all mortality in the experimental conditions was assumed to be due to predation by *A. helena* snails. No *A. helena* died during the trials.

After removal of snails that fed less than three times (n = 9), total consumption over the two week period of individual predators (feeding success) did vary significantly between treatments (*GLMM*_*COM* − *Poisson*_;*χ*^2^ = 16.319, *df* = 4, *P* = 0.003; Fig 2b). *A. helena* snails consumed more prey in the 2:1:4:1 treatment compared to the 1:3:2:2 (*z* = 3.165, *P* = 0.013) and 2:2:2:2 (*z* = 3.332, *P* = 0.008) treatment. The model with the best fit included ‘experimental day’ as random effect (*χ*^2^ = 29.87, *df* = 1, *P* < 0.001), besides the other random effects for predator individual (*χ*^2^ = 17.172, *df* = 1, *P* < 0.001) and position in the experimental tank (tank number: *χ*^2^ = 0.912, *df* = 2, *P* = 0.340; position in tank: *χ*^2^ = 1.341, *df* = 2, *P* = 0.512). Note that in 308 cases all individuals of one available prey species were consumed, but only in 31 (≈10%) of those cases another prey was eaten. In the latter *A. helena* may have been ‘forced’ to consume a non-preferred prey species.

### Selection of prey species

**Fig 3:**
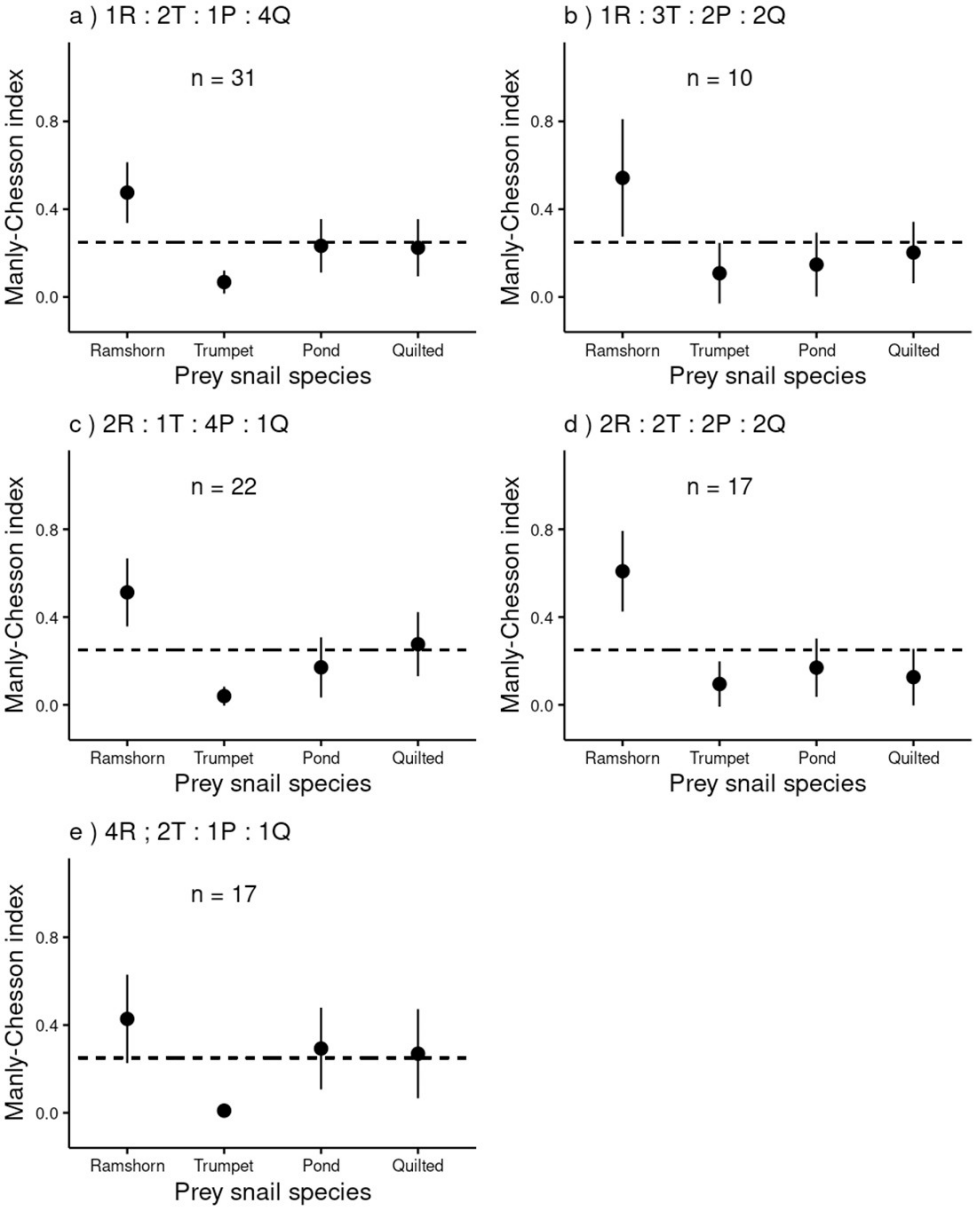
Prey selection in each treatment. Mean *α* (±95% CI) of each prey type in each treatment (a-e) based on feeding without depletion. The prey abundance in a particular treatment is indicated in the label of each panel (altogether 8 snails were used in each treatment). In the case where the confidence interval overlaps with the dashed line (expected feeding under random prey selection) there is no selective feeding on a prey species.

All species were consumed in all treatments (S1 Table), but Ramshorn snails were most consumed overall (Fig 3).

In the 1:2:1:4 (Quilted melania snails most abundant) treatment adding experimental day did not improve model fit (*χ*^2^ = 0.910, *df* = 1, *P* = 0.167). So the final model included predator ID and compartment nested within tank as random effects. There were overall differences in the consumption of the prey species (*GLMM*_*Poisson*_ *χ*^2^ = 183.84, *df* = 3, *P* < 0.001). Ramshorn snails were more consumed than the other species and Trumpet snails less consumed (Table 1). These species were also over and under consumed, respectively, compared to their abundance (Fig 3a).

**Table 1:** Comparison of consumption of prey types within treatments. Each row shows the *P*-value for the null hypothesis that there is no difference in consumption between the prey species. Results come from GLMM’s testing the likelihood of individual prey snails within treatments being consumed, based on species. Multiple comparisons were Tukey corrected within treatment. Prey species are Ramshorn snails (R), Malaysian Trumpet snails (T), Pond snails (P), and Quilted melania (Q) snails.

	estimate	z	*P*
1R:2T:1P:4Q			
Q—P == 0	-1.143	-6.007	<0.001
R—P == 0	0.770	4.133	<0.001
T—P == 0	-1.857	-6.630	<0.001
R—Q == 0	1.914	11.552	<0.001
T—Q == 0	-0.714	-2.676	0.0353
T—R == 0	-2.627	-9.954	<0.001
1R:3T:2P:2Q			
Q—P == 0	0.073	0.191	0.997
R—P == 0	1.692	4.944	<0.001
T—P == 0	-0.674	-1.639	0.354
R—Q == 0	1.619	4.830	<0.001
T—Q == 0	-0.747	-1.843	0.251
T—R == 0	-2.365	-6.426	<0.001
2R:1T:4P:1Q			
Q—P == 0	1.643	7.548	<0.001
R—P == 0	1.667	8.897	<0.001
T—P == 0	-0.648	-1.473	0.433
R—Q == 0	0.024	0.126	0.999
T—Q == 0	-2.290	-5.193	<0.001
T—R == 0	-2.314	-5.421	<0.001
2R:2T:2P:2Q			
Q—P == 0	-0.446	-1.231	0.5941
R—P == 0	1.384	5.277	<0.001
T—P == 0	-1.078	-2.427	0.0678
R—Q == 0	1.829	5.915	<0.001
T—Q == 0	-0.632	-1.335	0.5269
T—R == 0	-2.461	-6.123	<0.001
4R:2T:1P:1Q			
Q—P == 0	-0.214	-0.797	0.8378
R—P == 0	-0.532	-2.492	0.0503
T—P == 0	-4.389	-4.312	<0.001
R—Q == 0	-0.318	-1.403	0.4602
T—Q == 0	-4.176	-4.091	<0.001
T—R == 0	-3.858	-3.828	<0.001

In the 1:3:2:2 (Malaysian Trumpet snails most abundant) treatment the GLMM with the best fit also included experimental day as a random effect (*χ*^2^ = 6.700, *df* = 1, *P* = 0.010). Here, Ramshorn snails were more consumed than the other species, but there were no further differences between the consumption of the different prey types (Table 1). For all species the 95% CI overlapped with the expected value and can thus be considered consumed proportionally (Fig 3b).

In the 2:1:4:1 (Pond snails most abundant) treatment, no additional random effects were included in the best model (*χ*^2^ = 0.721, *df* = 1, *P* = 0.396). Ramshorn snails again were the most likely prey to be consumed, closely followed by Quilted melania snails (Table 1). Ramshorn snails were also over consumed compared to their abundance, and Trumpet snails were less consumed than expected under random feeding (Fig 3c; Table 1).

Under equal prey relative abundance, the 2:2:2:2 treatment, the different prey species were consumed at different rates (*χ*^2^ = 75.966, *df* = 3, *P* < 0.001; GLMM without additional random effects: *χ*^2^ = 0.674, *df* = 1, *P* = 0.412). Again, Ramshorn were more likely to be consumed than the other prey species, and between the other species there were no differences (Table 1). Like in the 1:3:2:2 treatment, the consumption of the prey species compared to random feeding, showed positive selection for Ramshorn snails and avoidance of Trumpet snails (Fig 3d).

In the 4:2:1:1 (Ramshorn snails most abundant) treatment, the best model did not include any additional random effects (*χ*^2^ = 0.406, *df* = 1, *P* < 0.524). Here, there were significant differences in consumption again (*χ*^2^ = 22.947*df* = 3, *P* < 0.001). Trumpet snails were consumed less than any other prey species and Pond snails were consumed less than Ramshorn snails (Table 1). Ramshorn were more consumed than expected under random selection (Fig 3e).

Overall, prey species appeared to be consumed by *A. helena* at consistent relative preferences, regardless of the relative abundances at which they were present. Ramshorn is always the most consumed prey and positively selected for (Manly-Chesson *α* ranging from 0.41 to 0.61) and Trumpet the least and negatively selected against (Manly-Chesson *α*: 0.01–0.14).

**Fig 4:**
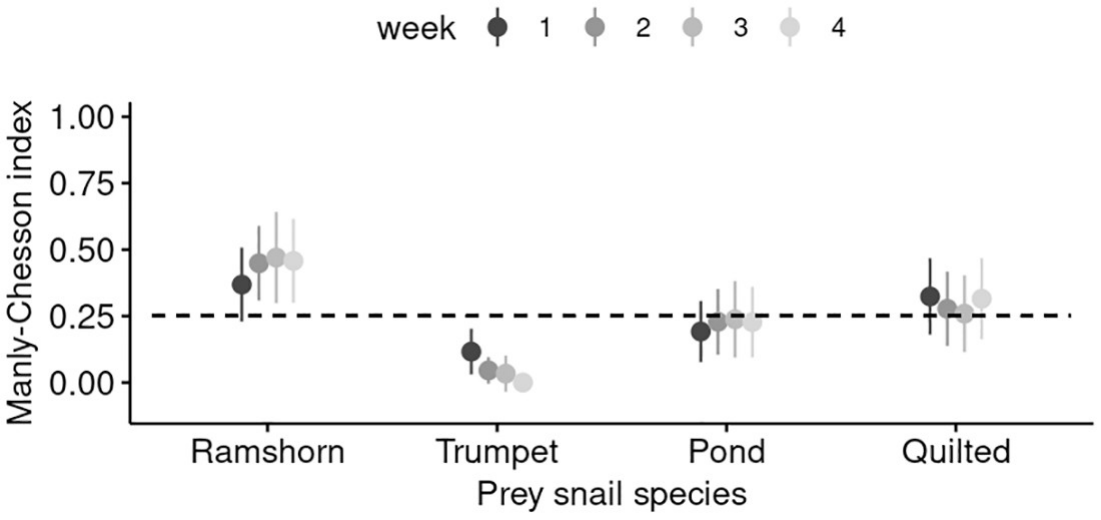
Weekly prey preference during the four week treatment. Preference of *A. helena* for each of the prey species in the four week feeding trial (prey composition: 1R:2T:1P:4Q). Mean values for all snails with ±95% CI. CI’s overlapping with the dashed line (expected consumption under random feeding per prey type) indicated no selection.

For the extended trials we found that overall, there were no differences between weeks in preference (i.e. no significant interaction between week and prey type; GLMM with predator ID as random effect, *z* < 1.573, *P* > 0.116; Fig 4) or individual selectivity of each predator (the corresponding data available in the analysis script). These extended trials also confirm that the feeding during the 14-day treatments is likely to be representative for the overall feeding of individual *A. helena* at this life stage.

### Selectivity and diet breadth of individual predators

**Fig 5:**
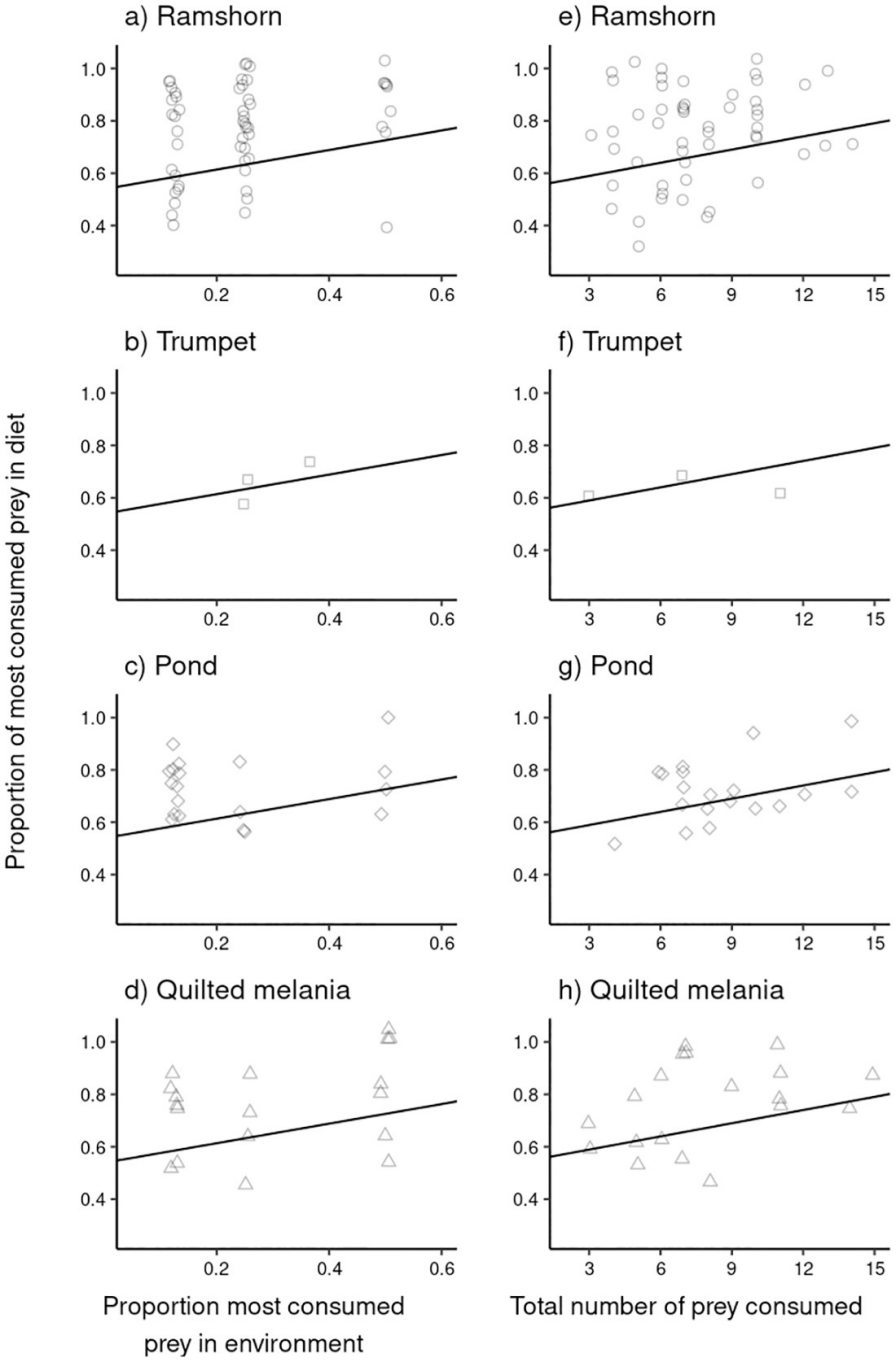
Individual preference of *A. helena* predators within the population measured in all feeding treatments. The left column (a-d) shows the relative abundance of the most consumed prey plotted against the abundance of the same type of prey in the environment. The solid black line shows the fitted model. Note that the fitted model is the same in all four subplots. The right column (e-h) presents the relative abundance of most consumed type of prey plotted against the total numbers of prey eaten. Again, the black solid line indicates the fitted model. In all graphs, each point corresponds to an individual *A. helena* predator. Individual data points are slightly jittered horizontally.

We explored the individual feeding preference of each *A. helena* snail. For simplicity, this is measured as the proportion of the most consumed prey in the diet across the duration of the experiment. Almost all *A. helena* demonstrated a high fidelity to a particular food source even at low densities of this prey type in the environment (Fig 5). The proportion of the most consumed prey in the diet increased with its abundance (GLMM *z* = 3.375, *P* < 0.001), and the total consumption (*z* = 2.947, *P* = 0.003). There were no differences between treatments (*χ*^2^ = 3.682, *P* = 0.451). We found that 94 out 97 *A. helena* snails had ≥50% of a particular prey in their diet (Fig 5). Moreover, the fidelity of an individual predator to a particular type of prey was observed across the full range of number of prey consumed by each *A. helena* snail (from 4 to 14).

**Fig 6:**
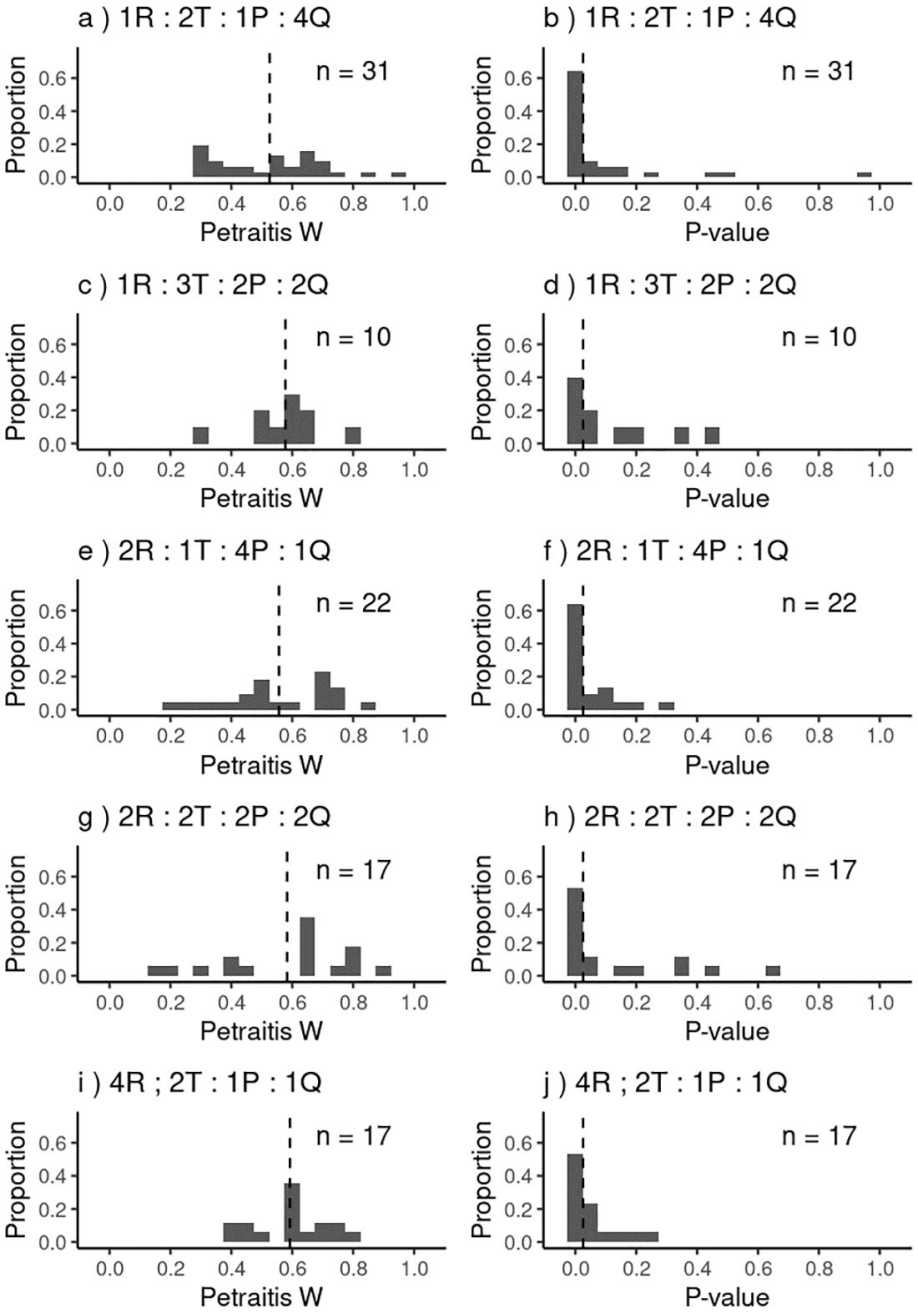
Histogram of *W*_*i*_-values with corresponding *P*-value distribution for *A. helena* in each treatment. Petraitis *W*_*i*_-values (left column) indicate the level of individual specialisation compared the the population diet. Higher values indicate a diet more similar to that of the population as a whole. The population mean deviation is indicated by the vertical dashed line. Histogram of *P*-values (right column), significant values (<0.05; left of the dashed vertical line) indicate a diet breadth narrow than that of the population, i.e. less variation in prey species consumed.

We further investigated whether individual predators actively selected for specific prey species. Many of the *P*-values (42 − 76%) associated with the calculated Petraitis’ *W*_*i*_ values were smaller than 0.05 and indicated that the diet breadth of an individual was narrower than that of the population (within a treatment; Fig 6). In all treatments at least a third of the individuals showed a individual diet breadth narrower than that of the population, and this proportion appeared to increase with increasing sample size.

## Discussion

Prey selectivity by individual *A. helena* snails was relatively consistent across treatments and time, with an overall preference for one of the prey species. Still, *A. helena* snails fed in a generalist manner at the population level showing a broad feeding niche including all prey types, but individual predators generally had a narrower feeding niche. Because feeding on different prey species varies consistently, we conclude that *A. helena* does distinguish between prey species.

In all treatments Ramshorn snails were significantly more consumed than other prey species by *A. helena* (Fig 2). Total consumption differed across the prey species, and treatments (Fig 3). However, because in all treatments all prey species were consumed, *A. helena* can still be considered a generalist predator [64], as reported earlier [32, 33]. There was a slight increase in consumption at higher relative abundance of the most consumed prey and total consumption of the predator (Fig 5). This is true across treatments as no differences between treatments were found. However, the overall preference for Ramshorn snails was stronger as even at low relative abundances they were often consumed more than expected under random feeding (Fig 3). Relative abundance independent feeding is observed in other systems as well [65–67] and this could represent a more common trait of generalist predator populations.

The selectivity by *A. helena* snails of their prey could be due to several factors, such as handling time [4], nutrient specific foraging [36, 37], learnt search images [68], or perceived profitability, as predicted by general foraging theory [4]. For instance, Trumpet snails were only sporadically consumed. This could be because of increased handling time when feeding on this prey species or (perceived) lower nutritional gains. The relatively small aperture of the Trumpet snails might make them difficult to consume for *A. helena*. In some predator-prey systems, prey activity contributes to explaining the higher electivity of predator towards this species, since the encounter rate increases even if the predator does not actively select for this prey [69, 70]. Here, Ramshorn snails may exhibit an ideal (from the predator’s point of view) activity pattern, i.e. to have a high encounter rate with the predator without being too fast and avoiding capture. The latter was possibly the case for Pond snails, which appear to move faster (to be further tested in future experiments).

In the one treatment further tested, there was temporal stability in feeding of *A. helena* (Fig 4). Not only the ranking of prey was consistent, but also the relative consumption appears fairly stable, especially after the first week. This indicates that the observed patterns in feeding behaviour are true patterns in feeding and not a short-term configuration of the population. It is however possible that over longer time periods these patterns shift and that, for example at different life stages, preferences change [11].

Interestingly, at the level of the individual predators, the patterns of feeding are more differentiated and well pronounced (Figs 5 and 6). Many individual *A. helena* snails have a significantly narrower diet breath than the population. These individual preferences persist over several weeks and individuals can be considered to be more specialised than the population as a whole. This pattern appears common amongst generalist species [64] and is for example seen in a number of spider species [71]. These spiders showed similar individual specialisation levels (*W* = 0.32 − 0.79) to *A. helena*. In coyotes, on the other hand, individual diets seem highly similar (mean *W* = 0.8), even between different social groups [72]. It is possible that the pack hunting of coyote allows them to employ more different predation strategies than individuals. However, this broadening of niche breadth is not seen in social spiders [71], it thus seems likely that other predator traits underlie the level of individual specialisation within a species. Levels of *A. helena* individual specialisation showed a wide range of values (Fig 6). For such predators one cannot easily define the functional response of the predator population as a whole, i.e. the food intake rate as the function of prey densities, as standard functional response formulations do not take into account individual preferences. It is unknown how the observed differences between individuals relate to feeding strategies. Further experiments would be needed to clarify the mechanisms that shape this food selectivity and the levels of flexibility in feeding.

Understanding the intraspecific heterogeneity of predator feeding behaviour is important because of its pronounced effects on community dynamics and biodiversity [73]. Structuring within the population of predators can cause spatial heterogeneity in the prey abundance if individual predators stay and feed on the same patch [74]. This then allows for predator niche separation within a species and reduction of interspecific competition, potentially resulting in larger population sizes [74]. Interspecific competition can also be affected by the variation in feeding within a species. If individuals within a species behave like specialist they are likely better competitors with other species and, thus, reduce the potential for coexistence with competing species [75]. This should also be taken into account when constructing mathematical models of such populations [76]. Furthermore, individual specialisation of predators can facilitate rapid adaptive speciation, including sympatric speciation [13].

Foraging theory of mixed feeding states that the growth and reproduction of generalist taxa is highest on a mixed diet [23, 37, 77–79]. This would indicate that *A. helena* might be selecting a suboptimal diet. However, if different prey types require different handling strategies, then learnt handling strategies could underlie individual feeding preference [16, 17, 80–82]. For example, although Trumpet snails might be difficult to consume, experience could lead to more efficient consumption of these snails. Learnt handing of a specific prey type could lead to high costs of switching between prey types for individual predators, which is unlikely to be compensated for by a wider range of nutrients from different prey types. In other words, regular switching to another prey type would reduce individual fitness [83], as seen in other systems [8, 77, 84]. Thus, one of the next steps will be to find out how feeding preferences in this species are shaped to gain insight into the costs and benefits of a mixed diet and prey switching.

In this study the same prey individuals were used multiple times within one batch of the same treatment to reduce the number of animals used [42]. This could have biased the results. Prey individuals within a species that were less palatable would be less likely to be eaten in repeated trials and so decreasing the overall consumption of this species. Given that prey individuals were not used in consecutive trials and the results are consistent between trials, this may have strengthened the observed effects, but not changing the overall patterns.

Empirical evidence of intraspecific feeding variation in predators has been seen in other animal taxa [14–16, 64, 74, 85]. We argue that *A. helena* presents an excellent additional biological model to explore diet specialisation across many prey types. It is also convenient to manipulate in the lab, can be used in long-term experiments, and allows accurate evaluation of prey items consumed. The ability to accurately evaluate consumption by counting empty prey shells and the fact that feeding and reproduction in both predator and prey happen at different time scales from feeding, make this system more amenable than, for example, copepod-plankton systems [86]. *A. helena* does also not store prey for later consumption as happens in some spider species [87]. However, the maximum daily consumption of each predator was only 2–3 prey items, making it less suitable for functional response experiments. *A. helena* snails appear only to feed on prey less than twice its own wet weight. Furthermore, recent work suggests that *A. helena* might be a species complex [28].

Future directions of research into trophic activity of freshwater predator snails should try to elucidate the mechanism of strong individual food selectivity by *A. helena*, including the role of the previous feeding experience. Also, mutual predator interference might occur, with different selectivity patterns when predators are housed together. Further, the diet breadth calculations would be particularly interesting for experiments with an even larger range of prey species.

To conclude, in this study, we found (i) that regardless of prey relative abundance feeding of *A. helena* was similar across treatments, and (ii) that feeding of individual predators was consistent over time. We further showed (iii) that most individual predators had a narrow feeding niche, whereas at the level of population, prey selectivity is less pronounced. Based on the above findings we conclude that (iv) *A. helena* can be a promising system to study relationships between individual and population diet niche breadth. Our results highlight apparent challenges in defining a generalist predator species depending on organisation level: individual or that of the whole population.

## Supporting information

S1 Fig Averaged prey selection in each treatment. Mean *α* (±95% CI) of each prey type in each treatment (a-e) based on feeding without depletion. The prey abundance in a particular treatment is indicated in the label of each panel (altogether 8 snails were used in each treatment). In the case where the confidence interval overlaps with the dashed line (expected feeding under random prey selection) there is no selective feeding on a prey species.
(PNG)

S1 Table Absolute consumption of all prey species per treatment. Note that the number of replicates differs per treatment, the number of replicates per treatment can be found in Fig 2.
(CSV)
