# Supplementary figures and images for: Assassin snails (Anentome helena) as a biological model for exploring the effects of individual specialisation within generalist predators
Source: PLoS One. 2022 Mar 14;17(3):e0264996. doi: 10.1371/journal.pone.0264996 (PMC8920249; doi:10.1371/journal.pone.0264996)

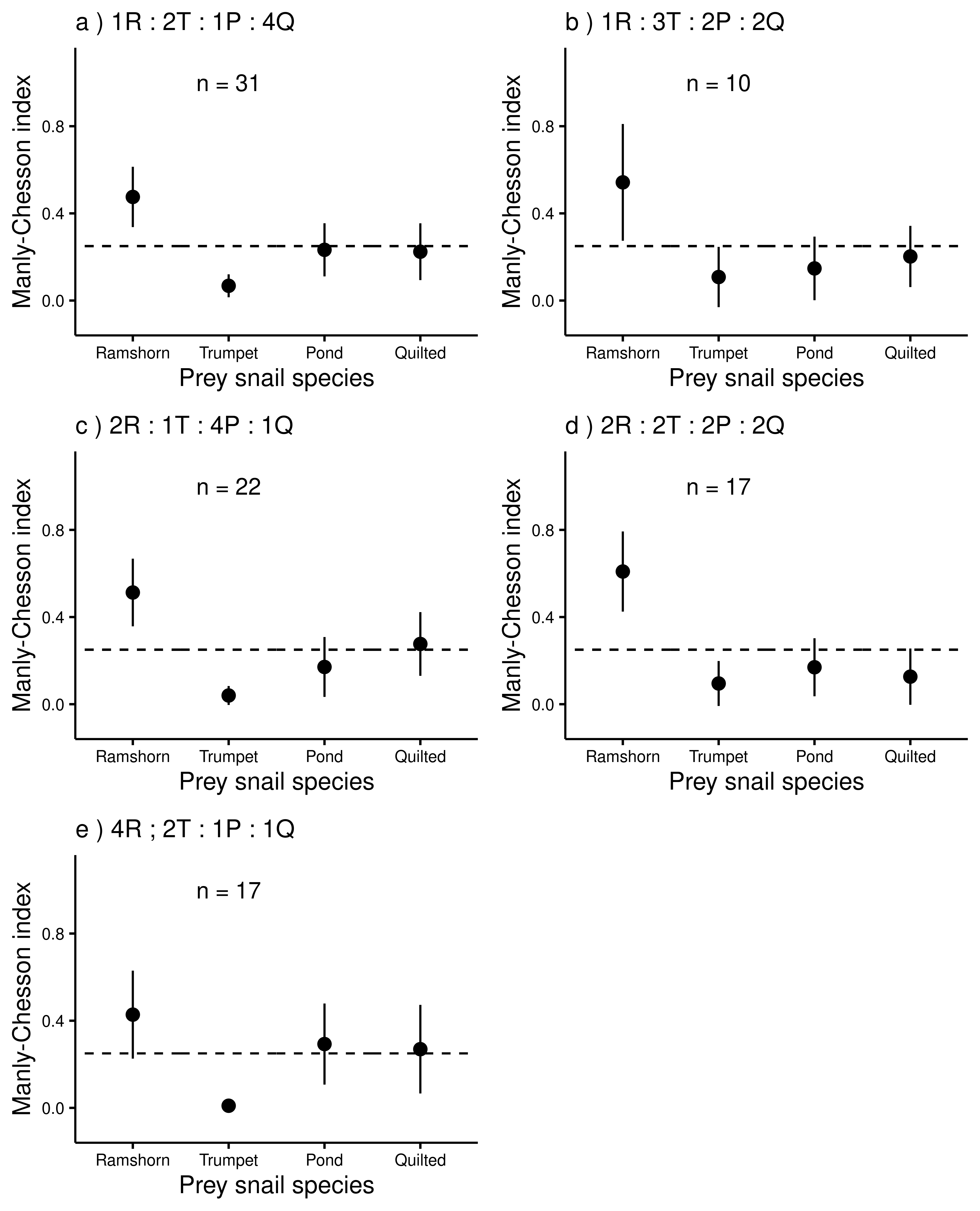

Supplement: S1 Fig — Mean α (±95% CI) of each prey type in each treatment (a-e) based on feeding without depletion. The prey abundance in a particular treatment is indicated in the label of each panel (altogether 8 snails were used in each treatment). In the case where the confidence interval overlaps with the dashed line (expected feeding under random prey selection) there is no selective feeding on a prey species. (PNG) [file pone.0264996.s001.png]
